# Chicago Medical Society; Meeting September 20

**Published:** 1886-11

**Authors:** 


### SOCIETY REPORTS.

---

CHICAGO MEDICAL SOCIETY:

*Stated Meeting, September 20, 1886.*—J. F. TODD, M.D., SECOND VICE-PRESIDENT, in the chair.

PROFESSOR N. S. DAVIS gave his

IMPRESSIONS OF THE BRITISH MEDICAL ASSOCIATION.

An abstract of his remarks is as follows:

The British Medical Association held its recent meeting at Brighton, the most fashionable and desirable watering place in England, very favorably situated for such a meeting of the profession of that country, being accessible to London as the great central point and having accommodations that probably cannot be found in any other town or city of moderate size in Great Britain. The British Association impresses one as being a body of men more advanced in life than the American Medical Association, not more intellectual development but more of what perhaps might be called sturdy physical development. Indeed this impression of solidity is forced upon the mind about everything.

The British Medical Association is not a representative body, in any sense, but was composed originally much after the fashion of calling together an assembly, organizing a national body and selecting a certain number to constitute a council and submitting the business affairs to the council. Subsequently a rule was adopted by the original council qualifying the profession in any given locality to form a local society and adopt such rules as were not in opposition to the constitution and by-laws of the national society; these societies were recognized as branches, and each member of a branch became nominally a member of the general body, with the right to attend the general meeting, but not entitled to take part in the general government. Each branch may elect one, two or three members of the council, so that as branch after branch has been organized, and the membership has increased, it has greatly enlarged the council, until, I gather from the proceedings of the Association, the council of the British Medical Association numbers seventy-one. This council has the entire control of the organization; they determine the places of meeting, and all matters in reference to the workings of the organization come before them first. Certain matters are brought before the general body at their meeting for their sanction, but they must originally go through the council. The council has at least four stated meetings in the year; they meet every quarter, and if business of importance is to come before them they may have other meetings. At the annual meeting of the Association the programme is so arranged that when they open at half-past twelve (they do not hold sessions in the forenoon), the council of the preceding year holds an hour's session. New members of the council are elected by the branches yearly, so that new members come in each year and others go out. Following this meeting, at two o'clock is the time for the meeting of the general body, but that did not occur this year; it was crowded out. At a subsequent hour we had what they call the council of the present year. The first meeting was the council of 1885, the next the council of 1886. The Sections held no meetings on the first day. I watched the proceedings carefully with special reference to learning what might be of use to our own National Association. One of the complaints made against us is that we lose too much time in eating and drinking, and social occasions, and too little advance is made in real science and professional knowledge.

The meeting of the council is held with closed doors. This peculiarity runs through everything. The council not only prepares the reports on business matters, but when they are submitted to the general body the council designates the man to move its adoption and the man to second the motion. I do not think that would be tolerated in America over one meeting, but it is a uniform, regular custom there and they seem to take to it very naturally.

The three sections that had the largest attendance were medicine, surgery and public medicine. They have the same trouble there as occurs in the American Medical Association, there is a certain number of members always coming and going. They come in and sit down, and if at a little distance they can't hear very well, they sit a little while and don't hear enough of the run of the subject under consideration to interest them and they go to another section, so that some are always moving about. Of the pathological and laryngological sections, one was held in the bedroom of King George and the other in what had been his china-closet, capable of seating not more than twenty or thirty people, and these seats were not all occupied. While the attendance does not exceed our attendance, there is about the same shifting and changing as at our general meetings. But the papers that were read had this characteristic to a greater degree than can be said of the papers as a whole with us, those who read papers had taken more pains to prepare them carefully, and to make them cover the ground fully. They had given more care to their preparation.

There is another feature of their meetings. Until the last three meetings of the American Medical Association there has not been any attempt to have parties informed of what particular papers are to be read in any of the sections, so that they might be prepared to lead off in the discussion of the papers. In 1883 in the Medical Section care had been taken to do this. Certain papers were to be read, and several members in different parts of the country were informed that a paper would be read on a certain subject and they were requested to lead in the discussion. The idea was to make the discussions of more value. This same course has been taken in a limited degree in our meetings since then. It is a general practice in the British Association. The officers of the sections, when they learn what papers are to be presented, judge as far as practicable who can impart interest, and have them informed of the general import of the paper and ask them to lead the discussion. Hence they elicit genuine benefit from the papers that are read and from the discussions that follow.

Many papers were prepared that were not read; undoubtedly they will find their way into print, as they do with us; the title was read and they were referred without anybody's knowing what they contained. Sometimes they are presented in abstract; the author thinks he will write it out, but fails to do so. I think in their work they are more deliberate, more complete; they exhaust a subject more nearly, and they endeavor to have the discussion so conducted as to elicit the most information from it. In studying their work as a whole, it seemed to me that there are some things we might copy with advantage.

Some members may be here who remember the proposition made in our own national organization years ago when the sections met and chose their officers. A proposition was made to change the by-laws so that the officers of the sections should consist of a president and secretary selected at each annual meeting for the next year, so that there would be officers already elected and the organization of the section would be perpetuated. I remember that the late Dr. Gross made a motion that, instead of having reports from standing committees on medical education, medical literature, and so on, which we had had previously, these committees be dropped and in their stead the association select at each meeting men supposed to be qualified for that especial work, in order to present an address to the general assembly, one on medicine, one on surgery, and one on obstetrics and diseases of women and children. That differed from the British programme only in substituting obstetrics and diseases of women and children for state medicine, but it was designed for the same purpose, to have one carefully prepared address read at each of the three last meetings, one on Wednesday, one on Thursday and one on Friday, while the president of the association would have an address on Tuesday especially designed for the general body. I seconded Dr. Gross' motion, but instead of its being carried, it was referred to a committee, as was also the proposition to have the officers of the sections appointed each year for the next, and at the next year the committee reported in favor of having the officers appointed for the following year. At the same time they reported in favor of having the president of each section present an address to the general body on the improvements in the section over which he presided. That was a substitute for Dr. Gross' motion. He and I opposed it on the ground that the presidents of the sections to be chosen each year ought to be selected from the older men, giving them an honorable position; but yet they would not be the men that would be likely to be adapted to read such papers. However, the proposition of the committee carried. Now we have seven or eight sections with seven or eight addresses before the general body, and what is the result? We find time the first day for the president's address, but the other days of the general meeting do not afford time enough for all the presidents to read their addresses; they are crowded off to the last day and one-third of the addresses prepared by the presidents of the sections are announced by title only. These addresses crowd the entire time of the general meeting of the body, and I think it would be a great deal better if we were to change our by-laws and appoint the first day for the president's address, which should be of a high order, covering the general field of medicine, another to surgery and another to obstetrics and gynæcology, or public medicine. Perhaps occasionally it would be as well to change the subjects for these addresses. The president of each section might deliver an address on any subject of interest to the section. This would insure a larger attendance and promote the work of the section, and yet would not embarrass the general meeting as it does when we try to get in seven or eight general addresses. That, I think, would be a real and substantial improvement in our body; one that we might copy with profit from the British association. Brief discussion of these addresses, and also of papers presented, might follow in some cases by members previously selected for it.

There has been a good deal said of late years about the work of the nominating committee in our National Association. This committee is appointed in a hurry by little knots gathered together who want to get through. The committee is hurried to do its business, and consequently the business is done very imperfectly. It has been claimed that we ought to have a more steady and conservative body, and the council of the British Association has been suggested as a pattern, but I do not think an organization like the British council would satisfy the medical profession of this country. I think we would not get along with it two years without rebellion against it. It seems to me, in studying the matter over, that we might remove the real evils we suffer from and yet not incur the evils that would follow from attempting to make a permanent council. It has been suggested that it be so arranged that one man would hold his place nine years. Instead of that, I think all the evils from which we are suffering could be obviated by appointing a business committee to consist of two members from each state in the union. Let each State Society that is represented in the organization be entitled to two members of that business committee and elect them the first year to hold office two years, and after they get started elect one each year, so that it would be permanent. By electing a new member each year it would be conservative and not totally changed. All the work that now devolves on the nominating committee could be done by this business committee, and if selected with proper care, it would be a convenient committee to which might be referred various propositions that come up, and which require careful consideration by members who represent the whole country.

There is an evil in the British council that is complained of now. They say this council has four meetings a year, and they have to meet in London because London is the centre of Great Britain. The practical working of that is that the profession within a radius of one hundred miles of London controls that council. It costs money to go from Edinburgh, Cork, Dublin or remote portions of England to London, and very few out of the whole number of seventy-one go. I learned that there were seven meetings held last year and thirteen of the members did not attend any of them. Not only that, but the more remote districts have found that their delegates so rarely attend, on account of the expense and loss of time, that half a dozen of them reported that they had not elected delegates. Now, if we were to appoint a permanent body, four-fifths of them would let their interest die out. Suppose a council should be organized that is to meet and transact business three times or even twice a year, how many would be found to go from the Pacific coast or from the north to a central point? It would not take ten years to gravitate so that your council would be in the hands of those within a hundred miles of the central point of meeting. If you made that Philadelphia or Washington or Chicago, the whole body would be ruled by a territory of two hundred miles, simply because parties would not incur the expense and spend their time to attend. They will not in Great Britain, and they are discussing the propriety of getting up some mode of compensating them to induce them to go. The plan I propose will, I think, after some degree of consideration, be found to obviate all the real evils we suffer from in hasty action and hasty nominating committees, and yet it would always secure us a satisfactory working body that would be reasonably representative of the several states, to attend to such things as are required to be attended to, and it would be a standing body of intelligent men to whom we could refer such things as would require consideration. The features that I have mentioned are characteristics that struck me as belonging to the British Association, which differ from ours.

In regard to the International Congress: Without solicitation, the council of the British Association altered the programme of one of their days, and announced that on Thursday at a certain hour the delegates from the American Association would be heard in regard to the International Congress. When the time came the hall was fuller than on any other occasion; the room was packed, and through the hallways and along the corridors, with people evidently having a decided interest in hearing what we had to say. As the other Americans insisted on my taking the lead, I went on the platform and occupied half or three-quarters of an hour in simple explanation. I made no allusion to anybody's differences except in general terms. We had had our troubles, and our frictions and our errors on all sides, but out of it all had come an organization truly national in its character, made up of delegates from all the states in the Union. This organization appointed an executive committee, to which had been transferred all further management of the Congress. This national committee had reported their work to the last meeting of the American Medical Association, and it was unanimously sanctioned, and so I felt justified in assuring them that this met the approval of the profession of the United States; and while we had no royal titles, the President of the United States, the heads of departments, and the President of the Senate had lent their names as patrons, and I assured them that they would receive as cordial a reception at that Congress as the American profession knew how to give. I would guarantee that they would be met by the intelligent part of the profession of every state in this Union from the Atlantic to the Pacific, and I cordially invited them to come. They received my invitation with the greatest degree of enthusiasm, and after a few remarks from Dr. Pancoast and others, which simply endorsed what I had said, they voted us their thanks and accepted our invitation to meet us in congress in 1887. Among some of the most prominent men in London and the provinces there is a warm zeal for attending the Congress. Three or four Americans have been on the continent for some time, and report that in France there is a genuine enthusiasm for attending the Congress. In France they do not pay the slightest attention to any differences we have, and just as far as they imagine there is coldness in England or Germany, the Frenchman comes with the more enthusiasm to make it up.

PROFESSOR W. E. CASSELBERRY read a paper entitled

PHARYNGEAL AND NASAL SURGERY BY THE GALVANO-CAUTERY; WITH REPORT OF CASES AND EXHIBITION OF APPARATUS, which appeared in the October number of the JOURNAL AND EXAMINER.

PROFESSOR F. O. STOCKTON, in opening the discussion, said he had listened with a great deal of pleasure to the paper; the cases are all very interesting. It does not seem necessary to spoil an electrode in order to make one for a special case; it seems that the ordinary flat electrode, by simply bending it down, would answer the same purpose. The trouble is, we must have new instruments for every case that comes up. Unfortunately, all practicing physicians, many of them who understand the throat and nose, are not so situated that they can run into an instrument maker's establishment at any time and have an electrode made, and it is wise to keep down to the standard instruments, which can be bought at any time and which are always at hand. Another point he desired to speak of is the idea that follicular pharyngitis and adenoid vegetations are one and the same thing. Some authors consider them the same, and others do not. Bosworth, of New York, claims that follicular pharyngitis and adenoid vegetations are one and the same. The battery which Professor Casselberry shows is one which Professor Stockton had used in practice for three years. He had tried a number of others, and found this one the most satisfactory. A short time ago a modification of it, which is a little better, was devised by Dr. Sajous, of Philadelphia, but it is not on the market at present. Many of our prominent writers have rather discouraged people from using the galvano-cautery, as they say it requires a great deal of skill, and knowledge not only of the anatomy of the part we are working upon, but also of electricity and the management of instruments. Dr. Morell Mackenzie, of London, makes that objection to the galvano-cautery; in fact, he says that all, or nearly all, the operations can be performed by simpler means. Dr. Cohen, in his last work, also refers to the difficulty of managing it, but if they had used the instruments of today they would not have found that difficulty.

DR. H. GRADLE said: The Doctor's paper was so complete there is nothing left for comment, except to deviate from the subject and take excursions into neighboring parts. It has been mentioned that a galvano-cautery is not absolutely necessary. That may be true as far the pharynx is concerned, but in the nose it is not. It would be almost impossible to do the same work as steadily by any other instrument, on account of the excessive hæmorrhage that would occur; these cases are unmanageable except with the cautery, and in most instances we can obtain relief both of local symptoms and obstruction of the nose as well as the nervous symptoms maintained by the excessive turgidity. The question was asked, whether hypertrophy of the pharyngeal tonsil could be identified with follicular pharyngitis; he should say not. We may have one or the other, but the follicles in the disease called follicular pharyngitis are much below the level of the gland. He has often found the gland hypertrophied into a very solid mass of about the consistency of the tonsils. The hypertrophy may of course have any extent; it may be so slight as to leave room for doubt of its existence, while in other cases it is impossible to gain any view of the posterior choanæ. In such cases nothing can be thought of but operation. He has used the cautery in the form of a white hot knife or hot snare for hypertrophy of the pharyngeal tonsil, but not satisfactorily. Where we have a distinctly pedunculated tumor it will do well enough to use the hot snare, but where the tonsil is hypertrophied into a solid mass not at all movable with the probe, he has found very little success with the battery, except that its removal is not so painful as by other means. He has at last settled down on the method suggested by Trautman, viz., a sharp curette, as sharp as the instrument-maker can make it, the shank of which is bent at an angle of 140°. The operation is a barbarous one as far as appearances go, because it requires force. There is pretty free hæmorrhage, but the pain is not very great. Unless the person is pretty tolerant it is not easy to remove the entire tonsil in one sitting, but in children he has not had any greater difficulty in coaxing them to the second sitting than at the first one. The results are extremely satisfactory, especially in cases of ear-disease due to this trouble. He has had considerable experience with different forms of battery; the kind which he prefers and recommends is an accumulator, if a wire can be had from some electric-light plant. He says he can speak well of the

McIntosh battery, but prefers one in which the carbon plates are made of the electric-light sticks. They are much harder than the ordinary carbon plates, and we can easily get a very large surface in the battery.

DR. R. TILLEY said he had had some experience with the cautery and with batteries and is now using the form of battery Dr. Gradle has referred to. He has the two cells attached so as to use them together, and have them so arranged that they can be lifted up with a crank; they are thus very easily manipulated. He has used this battery for about eighteen months, and it has always given satisfaction. He referred to a slight modification in the fluid which he has recently used; it consists in the use of bichromate of soda instead of bichromate of potash. The advantage is that when the fluid has been used for a considerable length of time, in using the potash there is always formed a large number of crystals in the bottom of the cell, which are very hard and require quite a little time to remove; and which are so closely connected with the cell that there is more or less danger of breaking the glass. In using soda instead of potash this does not occur. The compound thus formed is so soluble that no accumulation is formed at the bottom of the cell. He makes all of his own electrodes and uses, practically, wire alone. He sees no advantage in the knife over certain modifications of the wire. In operations in the post-nasal and pharyngeal regions he uses the cautery to burn the tissues completely to the bone. The object in view is to make a cicatricial tissue extending from the surface of the mucous membrane to the periosteum, so as to prevent that tumefaction which frequently occurs and fills up the nares. This tumefaction is quite remarkable sometimes and changes so readily that he has seen the anterior part of the turbinated bone swollen up and a moment afterwards the swelling gone, so that there could not be any possible reason or justification for using the cautery from the appearance of the tissues at the moment. Yet when the patient lies down at night he finds the nostril on the side on which he is lying so stuffed up that it is almost impossible to breathe. He has found that when the anterior part of the turbinated bone is burned well down to the bone tumefaction rarely occurs afterwards. Then there is a necessity of looking after these burns, especially in the posterior regions. In the anterior aspect of the nose there is only a small amount of mucus coming over the part that is burned, and no difficulties follow cauterization; but when the burned area is situated far back the mucus flows over it, and it not infrequently happens that unless the part is well attended to a certain amount of fungus growth develops in this region. He considers the after-treatment an important factor in cauterizations. He desired to say a word in regard to these adenoid growths in the posterior nares that Dr. Gradle has referred to. Dr. Trautman, of Berlin, removes all these vegetations with the sharp spoon, and claims remarkable success. Dr. Tilley has not seen anything like relatively the same number of cases in his practice that it would seem are met in practice in Berlin. He thinks adenoid growths are more rare in Chicago. With reference to their removal with the galvano-écraseur, he would be disposed to think that the ordinary wire écraseur would be more efficient than the sharp curette, and could be more easily manipulated and more of the growth taken off at the same sitting than with the galvano-cautery.

PROFESSOR W. E. CASSELBERRY, in closing the discussion, said, there were some points on which he wished to add a few words of explanation. In reference to spoiling an electrode in order to make a single loop to treat a special case, he spoiled the moxa-electrode, because it was an easier way to get the loop than to send to Philadelphia and have one made. As to the merits of the loop over a knife electrode, he thinks that in cauterizing the soft tissue, by taking a small elongated loop and pressing it into the part one gets a slough produced by each parallel wire, and it produces a larger slough with less pain than can be obtained by the knife electrode; the loop will sink further into the soft growth. If a plain knife electrode be used, the slough produced at once under the flat surface of the electrode prevents further effect from its caustic action. He had not only employed the small loop in this particular case, but had found it useful in cases of hypertrophy of the inferior turbinated body where it was unnecessary to cut off a large section by means of the knife or ècraseur. By simply taking the elongated loop and pressing it against the body, a considerable slough is produced in the center; after this has separated there remains an ulcer, and when the ulcer heals there is cicatricial tissue, which afterwards binds the body down.

In regard to the multiplicity of instruments, of course the multiplying of useless instruments is to be deprecated, but he would call to mind the fact that a large portion of the advance of medical science during the last half century has been directly due to the invention of new and improved instruments and apparatus. If those who devised the microscope, the ophthalmoscope, the laryngoscope, etc., had been satisfied with the "standard instruments" already in the market, we would not now have those most important forms of apparatus. A new instrument should always receive attention, and if it is a real improvement the inventor should be given due credit.

The glandular structures of the vault of the pharynx out of which adenoid vegetations are formed, and the follicles of the oro-pharynx, may not be histologically identical, but they are very similar in structure; they are both lymphoid tissues, and both exhibit the same tendency to undergo the hypertrophic process and to form similar large masses, which, if allowed to remain, produce morbid symptoms.

He did not know that it is necessary to say anything more in regard to batteries; there are a great many in the market. He is favorably impressed with the one which he uses and it has never failed to work. Although it may not be detrimental to have the plates remain in the fluid, yet, he said he has an uncomfortable feeling when he knows that his plates are being spoiled and the fluid used up faster than they need be. The Sajous battery spoken of is an improvement on the Fleming in this respect, the pedal is not so high from the floor. When one is operating, if he raises his foot to the height of eight inches he is liable to throw himself out of balance and to lose his line of vision. The pedal of the Sajous battery is only about two or three inches from the floor and it is unnecessary to raise the heel off the floor to work it, thus maintaining the balance of the body. The Sajous plates, also, are corrugated, thus giving the most surface in the least bulk. But unfortunately, the Sajous battery is not in the market. The one which Dr. Sajous uses he had made to order, but no doubt it will be put upon the market soon.

The various methods of operating for adenoid vegetations have all the same end in view, that is, the removal of the vegetations. The sharp curette is a good method if the patient will stand the blood, increase of pain and the length of the operation. In the case of children it is a rather difficult method, and requires considerable skill and fortitude on the part of the patient. The case related in this connection was a girl about fourteen years old. He first tried the forceps, but the pain in cutting off the adenoid vegetations was objected to very strenuously by the patient, and he was obliged to desist. Before he tried the hot snare he tried the cold snare, but found that it took some time to draw up the steel wire and thus squeeze off the vegetation. After the adoption of the hot snare he had no difficulty. The mere introduction of the instrument caused no pain, and after getting it over the vegetation, in two seconds the growth was off, with but little pain and no hæmorrhage to frighten the patient. The treatment of hay-fever by the cautery method, he would like to discuss, but the lateness of the hour did not permit it.

---

CHICAGO MEDICAL SOCIETY.

